# Electrophoretic Deposition of Multi-Walled Carbon Nanotube Coatings on CoCrMo Alloy for Biomedical Applications

**DOI:** 10.3390/mi14112122

**Published:** 2023-11-18

**Authors:** Bożena Łosiewicz, Patrycja Osak, Karolina Górka-Kulikowska

**Affiliations:** 1 Institute of Materials Engineering, Faculty of Science and Technology, University of Silesia in Katowice, 41-500 Chorzów, Poland; 2 Department of Biomaterials and Experimental Dentistry, Poznan University of Medical Sciences, 60-812 Poznań, Poland; karolinagorka.profil@gmail.com

**Keywords:** CoCrMo alloy, corrosion resistance, electrophoretic deposition, carbon nanotubes

## Abstract

Carbon nanotubes are a promising material for use in innovative biomedical solutions due to their unique chemical, mechanical, electrical, and magnetic properties. This work provides a method for the development of ultrasonically assisted electrophoretic deposition of multi-walled carbon nanotubes on a CoCrMo dental alloy. Functionalization of multi-walled carbon nanotubes was carried out by chemical oxidation in a mixture of nitric and sulfuric acids. The modified and unmodified multi-walled carbon nanotubes were anaphoretically deposited on the CoCrMo alloy in an aqueous solution. Chemical composition was studied by Fourier transform infrared spectroscopy. Surface morphology was examined by scanning electron microscopy. The mechanism and kinetics of the electrochemical corrosion of the obtained coatings in artificial saliva at 37 °C were determined using the open-circuit potential method, electrochemical impedance spectroscopy, and anodic polarization curves. The capacitive behavior and high corrosion resistance of the tested electrodes were revealed. It was found that the kinetics of electrochemical corrosion of the CoCrMo electrode significantly decreased in the presence of the functionalized multi-walled carbon nanotube coating. Electrophoretic deposition was shown to be an effective, low-cost, and fast method of producing nanotubes with controlled thickness, homogeneity, and packing density.

## 1. Introduction

Metallic biomedical materials should be durable and stable [1,2,3,4,5]. Their biocompatibility and corrosion resistance can be additionally enhanced by the use of multifunctional coatings covering the substrate surface, which support the treatment process [6,7,8,9,10,11,12]. Current work on this type of coating is very advanced and concerns micro- and nanotechnology solutions. At the end of the last century, the main focus was on research on thin layers and multilayer materials [6,7,8,9,12], but such work is still being undertaken today [13,14]. The beginning of this century was dominated by nanoparticles, and currently much attention is paid to nanowires, nanofibers, nanotubes, and hybrid materials [10,11]. Particularly interesting is the surface modification of metallic biomedical materials, developed in recent years, by creating porous layers of oxide nanotubes (ONTs) [11] or carbon nanotubes (CNTs) [15,16]. Such layers have been used as carriers in intelligent and controlled drug delivery systems, regenerative medicine, tissue engineering, personalized medicine, biosensors, or deep brain stimulation [16,17,18]. Moreover, CNTs can be obtained in the form of vertically arranged carbon nanotubes forming three-dimensional meshes. This method of obtaining CNTs is used to produce high-strength composites for aviation applications [19]. CNTs produced on the surfaces of materials increase their strength, in particular their resistance to cracking under dynamic loads [20]. MWCNTs are also used in glued-layer technologies. The use of CNTs obtained in the anodizing process increases the strength of the materials on the wall [21].

CNTs, as one of the allotropic varieties of carbon, are nanostructural materials with a length-to-diameter ratio above 1,000,000 [22]. These nanomaterials exhibit excellent mechanical and electrical properties. Their Young’s modulus is as high as 10^12^ Pa, which ensures elastic deformation and good tensile and bending strength. The unique properties of CNTs result from the presence of strong bonds between carbon atoms located in the graphite plane. CNTs are created by folding a monoatomic graphite plane. However, the folding method is not the same, which is why CNTs differ in length, diameter, and twist angle. CNTs are classified into single-walled carbon nanotubes (SWCNTs) and multi-walled carbon nanotubes (MWCNTs) [15,16,17,18,19,20,21,22,23,24]. SWNTs are flexible, while MWCNTs are stiff, rigid, and rod-like structures. MWCNTs are made of sp^2^ carbon. They take the shape of elongated and hollow cylinders with a relatively constant wall thickness along the axis and a straight internal channel. The ends of perfect MWCNTs are covered with semi-fullerene balls, which block access to the interior of the channel from the outside. Opening MWCNTs requires their removal by oxidation, milling, or ion beam treatment [22]. MWCNTs consist of several SWCNTs that are arranged in a coaxial Russian-doll structure. Each SWCNT corresponds to a single wall of MWCNT, and the wall-to-wall distance is 0.340 nm. The chemical properties of SWNTs and MWNTs are very similar, but due to the larger external diameter, MWCNTs are less reactive than SWNTs and, consequently, have less curvature and require functionalization.

Both SWCNTs and MWCNTs are obtained in the process of slow condensation of hot vapors of carbon atoms using the arc discharge, laser ablation, and catalytic chemical vapor deposition (CCVD) method [22,25,26,27]. Metals such as Co, Fe, Ni, and Pt, Si-based semiconductors, or porous metal oxides such as Al_2_O_3_ are usually used as catalysts [25]. Particularly important parameters of the CCVD synthesis process include the type of carbon source, such as hydrocarbons, alcohols, or CO, temperature, and pressure. Homogeneous catalysis is used, in which the carbon source and the catalyst are in a volatile form, or heterogeneous catalysis with the catalyst embedded in the solid phase. The advantages of the CCVD method include cost effectiveness, relatively low temperature, and continuous operation. The CCVD method produces aligned SWCNTs or MWCNTs that grow according to previously designed patterns, which ensures easy scalability and commercial application. The undoubted disadvantage of the CCVD method is the lower quality of the synthesized SWCNTs or MWCNTs caused by a large number of defects in the structure of the nanotube walls and the presence of carbon impurity deposits, as well as the need to remove the catalyst and/or its carrier in the post-synthetic purification stage. The electrophoretic deposition (EPD) method is used to obtain CNTs in the form of coatings on various metallic substrates, even with complex geometry, ensuring high purity of the obtained materials, which is difficult to obtain using other methods [28,29,30].

This work concerns shaping the functional properties of the biomedical CoCrMo alloy by creating, for the first time, a porous coating of MWCNTs on its surface by anaphoretic deposition in order to develop innovative dental material with increased corrosion resistance in the saliva environment. The choice of the CoCrMo alloy results from the fact that cobalt-based alloys are dental materials with good biotolerance, resulting from the presence of a self-passive layer on their surface, formed mainly by chromium(III) oxide [3,5]. CoCrMo alloys also show good resistance to pitting and crevice corrosion in chloride-containing solutions. Their mechanical properties and corrosion resistance are determined by their chemical composition and structure, which depend on the type of technology and manufacturing conditions.

## 2. Materials and Methods

### 2.1. Preparation of the CoCrMo Substrate

The metallic substrate was a dental CoCrMo alloy (BEGO GmbH & Co. KG, Bremen, Germany) containing 63.0 at.% Co, 30.0 at.% Cr, 5.0 at.% Mo, and the remaining components (Si, Mn, and C) below 2 at.%. The disc-shaped samples of 3 mm thickness were cut from cylinders of 6 mm diameter. Such obtained discs embedded in duracryl were first ground using 600 to 5000# SiC abrasive paper (Buehler Ltd., Lake Bluff, IL, USA). Then, mechanical polishing using the felt with a SiO_2_ suspension of 0.1 µm grain was conducted (OP-S, Struers Inc., Cleveland, OH, USA). A Forcipol 202 metallographic grinding and polishing machine (Metkon Instruments Inc., Bursa, Turkey) was used to prepare the one-sided mirror surface of the discs. The polished discs were sonicated twice in ultra-pure water (Milli-Q Advantage A10 Water Purification System, Millipore SAS, Molsheim, France) and acetone (Avantor Performance Materials Poland S.A., Gliwice, Poland) using a USC 300 TH ultrasonic cleaner (VWR International, Radnor, PA, USA) to remove the impurities after grinding and polishing.

### 2.2. Preparation of the MWCNT Suspension

Raw MWCNTs (MWCNTs-R) appearing as black powder of 10 g weight were synthesized using the CCVD method (M K Impex Corp., Mississauga, ON, Canada). Powder and MWNTs-R characteristics used for the EPD process are given in Table 1.

Before the EPD process, functionalization of MWNTs-R was carried out to introduce carboxylic acid groups (–COOH) by chemical oxidation in a 3:1 (*v*:*v*) mixture of concentrated HNO_3_ and H_2_SO_4_ (Avantor Performance Materials Poland S.A., Gliwice, Poland) at 100 °C for 4 h [31]. After cooling, the functionalized carbon nanotubes (MWNTs-F) were washed to neutrality with ultra-pure water. The functionalization carried out was aimed at ensuring better dispersion of MWCNTs-F with negative charges. The aqueous (aq) suspension of 0.5 mg mL^−1^ MWCNTs-F was prepared for the EPD process using ultrasonication at a frequency of 45 kHz and a power of 200 W for 15 min, and centrifugation at 3000 rpm for 15 min. For comparative purposes, an aq suspension of MWCNTs-R was also prepared.

### 2.3. Electrophoretic Deposition Conditions of the MWNT Coating

**Figure 1:**
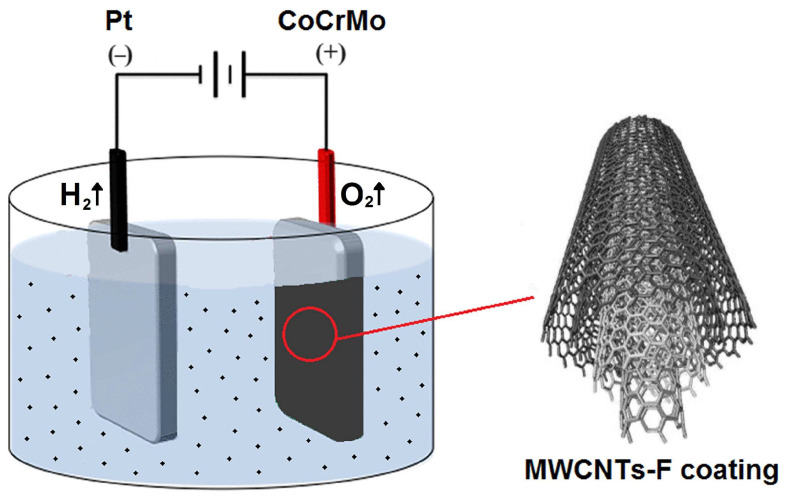
Schematic illustration of the electrophoretic deposition setup of the MWCNTs-F coating over CoCrMo substrate from a colloidal suspension.

Ultrasonically assisted EPD was performed in a two-electrode system containing a platinum cathode (S = 8.0 cm^2^) and an anode made of CoCrMo alloy (S = 0.5 cm^2^). The method of preparing CoCrMo electrodes was described in a previous work [32]. The cathode and anode were placed face-to-face with each other at a fixed distance of 1.5 cm. A schematic of the EPD setup is illustrated in Figure 1.

During the colloidal process, the negatively charged MWCNTs-F were directly deposited onto the CoCrMo substrate from a stable suspension under an electrical field. A colloidal suspension of the MWCNTs-F and ultra-pure water used as a solvent was sonicated. An ultrasonic cleaner produced a high shear force, leading to the exfoliation of MWCNT bundles and the production and bursting of air bubbles, ensuring the homogeneity of the suspension. Setting the solution aside for several hours improved the dispersion of MWCNTs in ultra-pure water. Such treatments prevent large concentration drops, regardless of the type of CNTs. Using this method, a stable solution of MWCNTs in ultra-pure water was obtained without the need to functionalize the use of surfactants as a component of the solution [33,34].

In the aq solution used, the water electrolysis took place with oxygen gas evolution at the anode surface, according to Reaction (1):

(1) $$2H_{2}O\to O_{2}↑+4H^{+}+4e^{-},$$

and hydrogen gas evolution at the cathode surface, as described by Reaction (2):

(2) $$4H_{2}O+4e^{-}\to 4OH^{-}+2H_{2}↑.$$

The anaphoretic deposition process was conducted at a voltage of 20 V for 5 min at room temperature using a PWR800H high-current power supply (Kikusui Electronics Corporation, Yokohama, Japan). For comparison, the MWCNTs-R coating was also deposited over the CoCrMo anode. After the EPD process, the anodes were dried in air at room temperature.

### 2.4. FE-SEM Study of the MWCNT Coatings

The microstructure and thickness of the obtained MWCNT coatings were examined by scanning electron microscopy with field emission (FE-SEM) using a Hitachi HD-2300A scanning electron microscope (Hitachi Ltd., Tokyo, Japan) at an accelerating voltage of 15.0 kV. A magnification of 20,000 and 50,000 times was used in the microscopic observations made. The samples did not require preliminary surface preparation.

### 2.5. ATR-FTIR Study of the MWCNT Coatings

The functional groups of the MWNTs-R powder and MWNTs-F coatings were determined by the attenuated total reflectance–Fourier transform infrared spectroscopy (ATR-FTIR) method. An IR Trace-100 spectrophotometer equipped with an ATR attachment with a diamond crystal (Shimadzu, Kyoto, Japan) was applied for the collection of the ATR-FTIR absorption spectra. The measurements were performed in the spectral region of 4000–400 cm^−1^ at a rate of 0.2 cm s^−1^, and a resolution of 4 cm^−1^. Each obtained spectrum was averaged from 100 interferograms. Before collection, the atmospheric background was recorded under the same measurement conditions and then automatically subtracted from the spectra of the tested samples.

### 2.6. Corrosion Resistance Study of the MWCNT Coatings

The corrosion resistance of the MWCNTs-F coatings on the CoCrMo substrate was studied in vitro in deaerated AFNOR artificial saliva at pH = 7.4(1) at 37(2) °C [35]. For the pH adjustment of the solution, 4% NaOH and 1% C_3_H_6_O_3_ were used [36]. Ultrapure water with a resistivity of 18.2 MΩ cm was used as the solvent. All reagents used were pure for chemical analysis (Avantor Performance Materials Poland S.A.).

The electrochemical cell contained the working electrode as a sample under study, the counter electrode as a Pt foil, and the reference electrode as a saturated calomel electrode (SCE) with a Luggin capillary. The Autolab/PGSTAT12 (Metrohm Autolab B.V., Utrecht, The Netherlands) was used for direct current (DC) and alternating current (AC) measurements. Firstly, the open-circuit potential (E_OC_) was recorded for a time (t) of 2 h according to the ISO 10271:2021 standard [36]. Next, electrochemical impedance spectroscopy (EIS) spectra were collected at the E_OC_ in the frequency (f) range of 10 kHz–1 mHz with a sinusoidal signal amplitude of 10 mV. The EQUIVCRT program and the complex non-linear least squares (CNLS) method were used to analyze the obtained EIS spectra. The description code of equivalent electrical circuits by Boukamp was applied [37]. Finally, the anodic polarization curves were recorded from a potential of 150 mV more negative relative to the Eoc up to 1 V with a polarization rate of 1 mVs^−1^.

## 3. Results and Discussion

### 3.1. Microstructure Characteristics of the MWNTs Coatings

**Figure 2:**
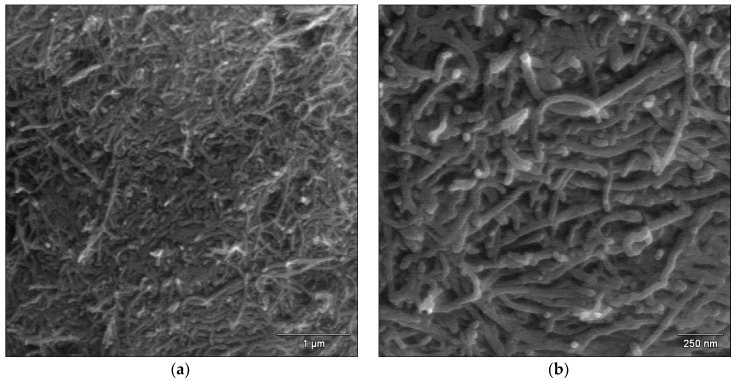

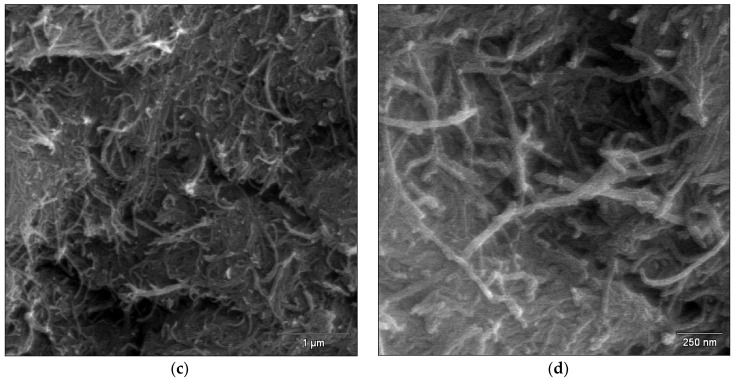
FE-SEM image of the CoCrMo alloy after the EPD process at 20 V for 5 min in a colloidal suspension containing: (**a**,**b**) MWCNTs-R; (**c**,**d**) MWCNTs-F.

Microscopic analysis was used to characterize the microstructure of the obtained MWCNTs coatings on the surface of the dental CoCrMo alloy. Scanning electron microscopy is an indispensable tool that enables the proper characterization of the size and shape of carbon nanotubes [38,39,40,41]. Figure 2 presents exemplary FE-SEM images showing the surface morphology of MWCNTs-R and MWCNTs-F coatings with a thickness of 7.54 ± 0.55 µm and 6.89 ± 0.32 µm, respectively.

The obtained MWCNTs-R coatings showed poor adhesion to the alloy substrate (Figure 2a,b). The FE-SEM images show the lack of MWCNTs-R aggregates and the even distribution of the embedded nanotubes over the entire surface of the substrate in the observed micro-area. The outer mean diameter of MWCNTs-R used was 30–50 nm, and the length of the nanotubes used was from 0.5 to 2 μm (see Table 1). The MWCNTs-R coating is characterized by the presence of longer-length nanotubes and tighter packing, with a tendency to form nanotube bundles compared to the MWCNTs-F coating (Figure 2c,d). The obtained MWCNTs-F coatings showed stronger adhesion to the metallic substrate. Analysis of FE-SEM images of the surface morphology of the MWCNTs-F coating shows no damage to the deposited carbon nanotubes despite their chemical oxidation in the functionalization process in a mixture of strong acids. MWCNTs-F became shorter and more coiled as a result of functionalization. The deposited MWCNTs-F do not create characteristic bundles, as in the case of MWCNTs-R. Similar behaviors of functionalized MWCNTs have been observed in the literature [38,39]. It should be noted that carbon particles in carbon nanotubes are connected by weak Van der Waals forces [40]. The functionalization process causes the C=C bond to be broken, as a result of which the MWCNTs-F are shortened [39]. Chemical oxidation of MWCNTs-R in the mixture of HNO_3_ and H_2_SO_4_ results in the formation of carboxyl groups, which increases the ability of the thus obtained MWCNTs-F to absorb water inside the nanotubes and reduces their toxicity [41]. MWCNTs-F also show a more stable structure compared to MWCNTs-R.

In the presented work, a short ultrasonic treatment time was used to improve the dispersion of carbon nanotubes in ultrapure water, which was only 15 min. The time of ultrasound treatment was selected based on literature reports, according to which the best dispersion effect of carbon nanotubes is achieved when the ultrasonic exposure time does not exceed 60 min [42]. If longer times are used, the structure of carbon nanotubes is destroyed, mainly as a result of ultrasonic cavitation and the chip effect. According to microscopic observations, the shortening of the carbon nanotubes used occurred as a result of functionalization in a mixture of acids.

### 3.2. Functional Groups Characteristics of the MWNTs Coatings

**Figure 3:**
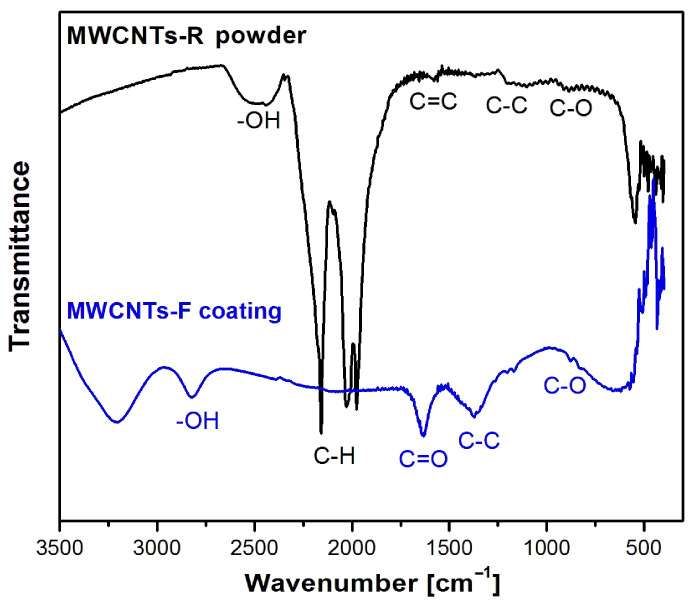
ATR-FTIR spectra collected for MWCNTs-R powder and MWCNTs-F coating deposited over CoCrMo alloy.

ATR-FTIR is a commonly used qualitative method for determining the functional groups of tested materials. Figure 3 shows a comparison of the ATR-FTIR absorption spectra collected for MWCNTs-R powder (black line) and MWCNTs-F coating deposited over CoCrMo alloy (blue line) using the EPD process.

Analysis of the FTIR spectrum obtained for the starting MWCNTs-R powder showed the presence of a peak at 1579 cm^−1^, which is attributed to the stretching bonds of the C=C carbon bond. The band at 1520 cm^−1^ is associated with the vibrational vibrations of the C–C bond, and the peak at 1230 cm^−1^ is associated with the stretching vibrations of the C–O bond. The band in the range of 2350–2650 cm^−1^ corresponds to the bonds of the O–H group associated with the presence of amorphous carbon [43]. The peaks located at 2032 cm^−1^ and 2158 cm^−1^ are assigned to the asymmetric stretching of aCH_2_ and symmetric sCH_2_ of the C–H bond [44].

The ATR-FTIR absorption spectrum obtained for the CoCrMo alloy with a MWCNTs-F coating confirmed the functionalization of MWCNTs-R and the deposition of functionalized carbon nanotubes by ultrasonically assisted EPD. The presence of a broad peak at approximately 2995 cm^−1^ was revealed, which refers to the stretching of the hydroxyl group O–H coming from carboxyl groups (O=C–OH and C–OH). The presence of this group is related to the breaking of the van der Waals bonds present in the MWCNTs-R powder. The peaks occurring at 1641 cm^−1^ and 1058 cm^−1^ are characteristic of multi-walled carbon nanotubes. This band is associated with the occurrence of stretching vibrations corresponding to C=O and C–O bonds of carboxyl groups –COOH [38].

### 3.3. Open-Circuit Potential Measurements

**Figure 4:**
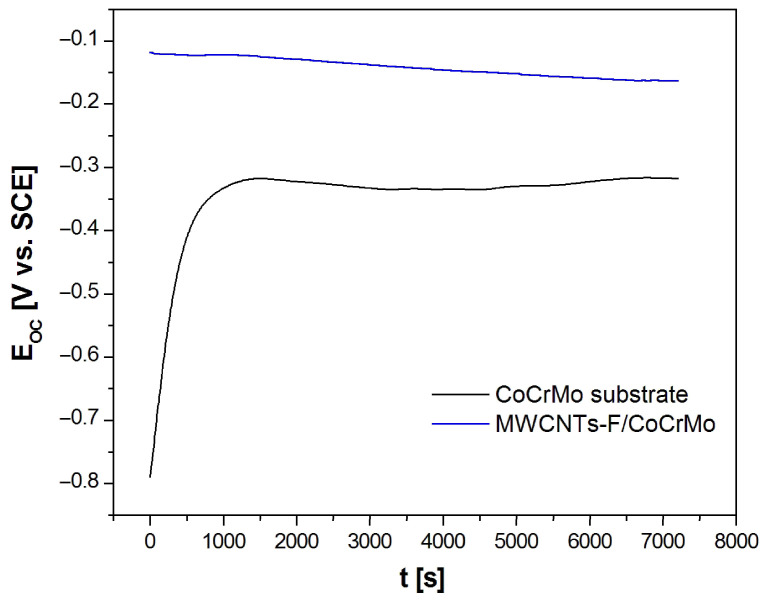
Dependence of open-circuit potential (E_OC_) on time (t) for the CoCrMo electrode before and after deposition of MWCNTs-F coating in artificial saliva at 37 °C.

A preliminary assessment of the influence of the obtained MWCNTs-F coating on the in vitro corrosion resistance of the CoCrMo alloy in artificial saliva at 37 °C was carried out using the open-circuit potential method. Figure 4 shows the results of E_OC_ measurement as a function of t for the CoCrMo electrode before and after deposition of MWCNTs-F coating. Different courses of the obtained E_OC_ = f(t) curves are observed, which indicates various corrosion behaviors of the tested electrodes. In the case of the CoCrMo electrode, during the first 15 min, the E_OC_ value increases dynamically from −0.790(67) V to −0.320(48) V, and then stabilizes at approximately −0.317(47) V. This course of the E_OC_ = f(t) curve is typical for metallic electrodes self-passivating in aq solutions and indicates an increase in the corrosion resistance of the electrode as a result of the formation of a barrier oxide layer on its surface, which seals over time and protects the metallic substrate against corrosion [6,7]. The tested CoCrMo alloy contains as much as 30.0 at.% Cr, which is capable of self-passivation.

The rate of E_OC_ changes for the CoCrMo electrode with the deposited MWCNTs-F coating is much slower than for the alloyed substrate. The E_OC_ value in the presence of the MWCNTs-F coating decreases slightly from −0.118(18) V to approximately −0.160(22) V within 6500 s, and then reaches a stable value at −0.163(24) V. This nature of E_OC_ changes results from the presence of the porous MWCNTs-F coating and the penetration of electrolyte into the open pores. However, it should be emphasized that the CoCrMo electrode with the MWCNTs-F coating shows an E_OC_ value almost twice as high as the CoCrMo electrode, which proves the impact of the surface modification on improving the corrosion resistance of the tested dental alloy. The E_OC_ values determined after 7200 s of immersion were considered to have approximate corrosion potential (E_cor_) in further electrochemical measurements.

### 3.4. Electrochemical Impedance Spectroscopy Measurements

AC measurements were carried out to study the effect of the MWCNTs-F coating on the mechanism and kinetics of electrochemical corrosion of the dental CoCrMo alloy in artificial saliva. The explanation of the impedance behavior of the tested materials was based on the well-known concept of equivalent electrical circuits. To improve the CNLS fitting, the capacitor was replaced with a constant-phase element (CPE), which represented a “leaky” capacitor with non-zero real and imaginary components. The impedance of the CPE (${\hat{Z}}_{CPE}$) is defined by Equation (3) [45]:

(3) $${\hat{Z}}_{CPE}=\frac{1}{T{\left(j\omega \right)}^{\varphi }}$$

where T is a capacitive parameter in F cm^−2^ s^ϕ−1^ depending on the electrode potential, and ϕ is the rotation angle of the purely capacitive line on the complex plane plots, α = 90° (1 − ϕ).

**Figure 5:**
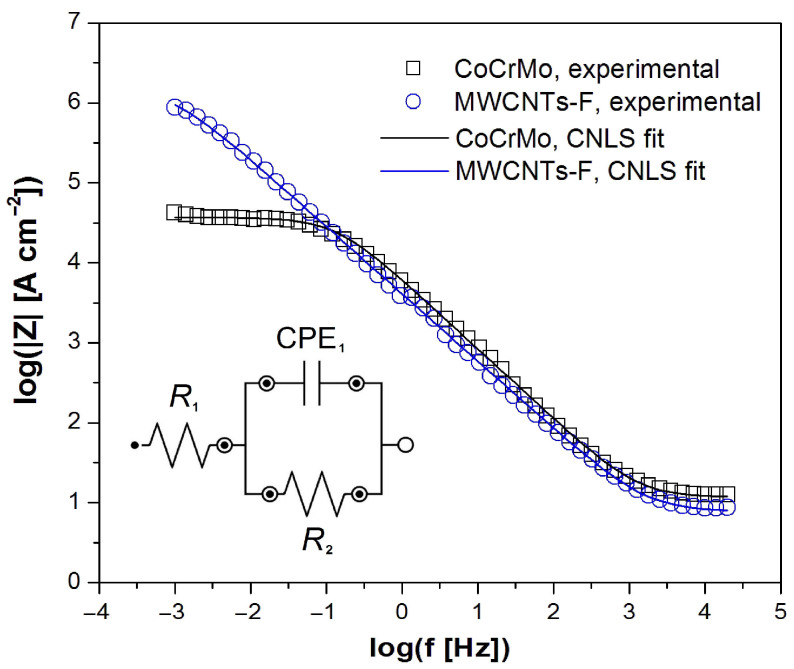
Experimental (symbols) and fitted (continuous lines) Bode diagrams of log|Z|=f(log f) obtained at the E_OC_ for the CoCrMo electrode before and after deposition of MWCNTs-F coating in artificial saliva at 37 °C.

All experimental EIS spectra were analyzed using the CPE1 model shown as an inset in Figure 5. The CPE1 model with a one-time constant in the electrical circuit consisted of a resistor (R_1_), which was connected in series with one parallel CPE_1_-R_2_ system. This model produces only one semicircle on the complex plane plot [6,45]. The R_1_ resistor is associated with the solution resistance. CPE_1_ denotes the electrical double-layer capacity. The R_2_ resistor is the charge transfer resistance (R_ct_) through the interface of the electrode and solution.

**Figure 6:**
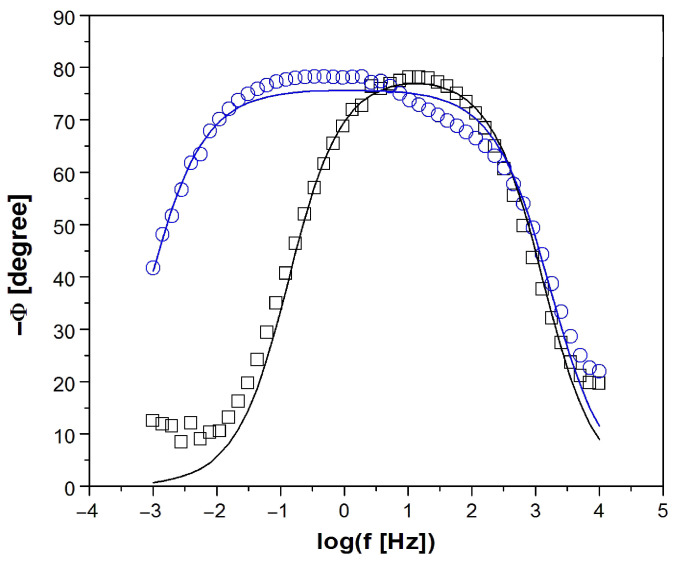
Experimental (symbols) and fitted (continuous lines) Bode diagrams of phase angle (Φ) versus frequency logarithm (log f) obtained at the E_OC_ for the CoCrMo electrode before and after deposition of MWCNTs-F coating in artificial saliva at 37 °C.

The Bode diagrams in Figure 5 and Figure 6 present the experimental (symbols) and CNLS-fitted data using the CPE1 model (continuous lines) for the electrochemical corrosion of the CoCrMo electrode without and with deposited MWCNTs-F coating in the artificial saliva solution at 37 °C. A very good quality of the CNLS fit between the simulated EIS spectra and the experimental data was found. In the Bode diagrams of log|Z| = f (log f) shown in Figure 6, the slope in the medium frequency range is close to −1. There is also a large difference in the log|Z| values in the lowest frequency range, which indicates a significant improvement in the corrosion resistance of the CoCrMo electrode after surface modification was applied. The value of log|Z|at f = 1 mHz is equal to 4.63(52) and 5.94(61) Ω cm^2^ for the CoCrMo electrode and MWCNTs-F coating over the CoCrMo electrode, respectively.

Bode diagrams shown in Figure 6 are characterized by maximum values of the phase angle of about −80° for both types of electrodes studied. However, in the case of the CoCrMo electrode with the deposited MWCNTs-F coating, a wider plateau is visible in the medium frequency range, which indicates its higher corrosion resistance compared to the unmodified CoCrMo electrode. High values of |Z|_f→0_ in Figure 5 and Φ in Figure 6 testify about the capacitive behavior of the CoCrMo electrode before and after surface modification.

Table 2 shows the average values of R_1_, CPE_1_-T, CPE_1_-ϕ, and R_2_ parameters, which describe the equivalent electrical circuit displayed in Figure 5 used for the CNLS fitting procedure of experimental EIS spectra recorded at the E_cor_ for the CoCrMo electrode before and after surface modification in the artificial saliva at 37 °C.

The CPE1-T parameter for both electrodes has values of the order of 10^−5^ F cm^−2^ s^ϕ−1^, which are typical for metallic biomaterials in the environment of physiological saline and body fluids [6,45]. The slightly higher CPE1-T value for the modified CoCrMo electrode may be due to its larger surface development compared to the alloy substrate. The CPE_1_-ϕ empirical parameter in both cases takes values much smaller than 1, and is due to the physical, chemical, and geometrical heterogeneities on the electrode surface [45]. The R_2_ kinetic parameter for the MWCNTs-F/CoCrMo electrode is 1.61(16) × 10^6^ Ω cm^2^. This value is two orders of magnitude higher compared to the unmodified CoCrMo electrode and proves the significant effect of the surface modification used on the increase in its corrosion resistance. The average values of parameters obtained as a result of the CNLS fitting prove that the self-passive oxide layer on the surface of the CoCrMo electrode has weaker barrier properties in the artificial saliva solution compared to the CoCrMo electrode with the deposited MWCNTs-F coating.

### 3.5. Potentiodynamic Characteristics

**Figure 7:**
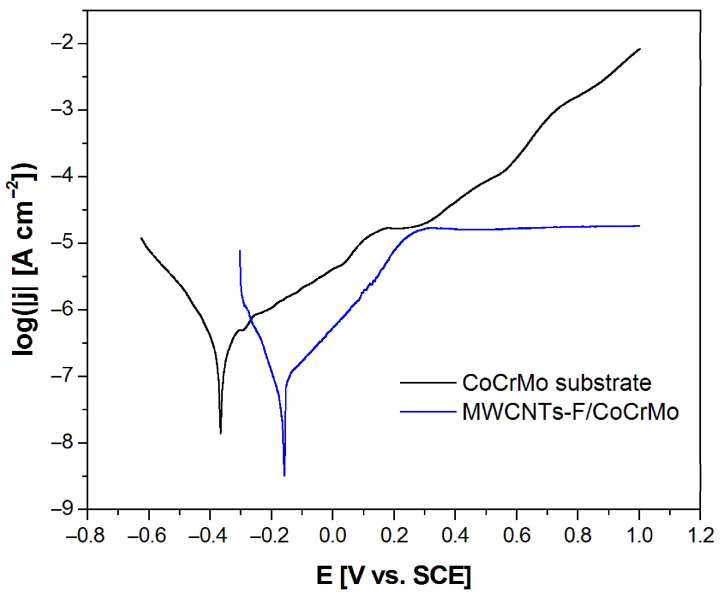
Anodic polarization curves of the CoCrMo electrode before and after deposition of the MWCNTs-F coating obtained at a polarization rate of 1 mV s^−1^ in artificial saliva at 37 °C.

Figure 7 shows the anodic polarization curves in a semi-logarithmic scale obtained for the CoCrMo electrode before and after deposition of MWCNTs-F coating in artificial saliva at 37 °C. It is visible that the modification of the CoCrMo electrode surface resulted in a significant shift of the log|j| = f(E) curve towards positive potentials, which indicates an increase in the corrosion resistance of the CoCrMo electrode. The E_cor_ increased from −0.365(47) V for the CoCrMo electrode to −0.157(26) V for the MWCNTs-F coating deposited on the alloy substrate. Simultaneously, the corresponding current density (j_cor_) value decreased from 1.25(32) × 10^−8^ A cm^−2^ to 2.04(22) × 10^−9^ A cm^−2^. The obtained values of E_cor_ and j_cor_ are typical of metallic biomaterials in the biological milieu [6].

In the range of potentials corresponding to the cathode branch below E_cor_, the tested electrodes show resistance to electrochemical corrosion. At potentials above the E_cor_, anodic dissolution takes place. However, it should be emphasized that in the case of the surface-modified CoCrMo electrode, at potentials above 0.3 V, passive behavior and protection of the substrate against pitting corrosion are visible, which is ensured by the presence of the MWCNTs-F coating.

## 4. Conclusions

The surface modification of the CoCrMo dental alloy containing 63.0 at.% Co, 30.0 at.% Cr, 5.0 at.% Mo, and the remaining components (Si, Mn, and C) below 2 at.% by the ultrasonically assisted EPD of the MWCNTs-F coating under the proposed conditions leads to a significant increase in the corrosion resistance in the artificial saliva. Quantitative assessment of electrochemical corrosion resistance based on EIS measurements allowed for the characterization of the impedance of the interface of the electrode and artificial saliva, which can be described using the equivalent electrical circuit of the CPE1 model. AC impedance characteristics revealed the capacitive behavior and high corrosion resistance of the tested materials. Potentiodynamic characterization showed passive behavior and reduced susceptibility to pitting corrosion in the presence of chloride anions for the modified CoCrMo electrode. The obtained results confirmed that the CoCrMo alloy with the deposited MWCNTs-F coating meets the pitting corrosion resistance criteria for dental materials.

## Figures and Tables

**Table 1:** Characteristics of MWNTs-R used for the EPD process.

**Powder Characteristics**	Apparent density	50–150 kg m^−3^
	Mean agglomerate size	200–500 μm
	Weight loss at 105 °C	<1%
**MWNTs-R Characteristics**	C content	>90 wt.%
	Free amorphous carbon	Not detectable (SEM/TEM)
	Mean number of walls	5–15
	Outer mean diameter	30–50 nm
	Length	0.5–2 μm

**Table 2:** The results of the CNLS fitting of the experimental EIS data for the CoCrMo electrode and the CPE1 model for the electrochemical corrosion in the artificial saliva at 37 °C (see Figure 5).

Electrode	R_1_ (Ω cm^2^)	CPE_1_-T (F cm^−2^ s^ϕ−1^)	CPE_1_-ϕ	R_2_ (Ω cm^2^)
CoCrMo	11.72(92)	0.74(9) × 10^−5^	0.880(14)	3.71(21) × 10^4^
MWCNTs-F	7.89(19)	0.82(9) × 10^−5^	0.841(11)	1.61(16) × 10^6^

## Data Availability

Data are contained within the article.
